# Interaction of the Coronavirus Infectious Bronchitis Virus Membrane Protein with β-Actin and Its Implication in Virion Assembly and Budding

**DOI:** 10.1371/journal.pone.0004908

**Published:** 2009-03-16

**Authors:** Jibin Wang, Shouguo Fang, Han Xiao, Bo Chen, James P. Tam, Ding Xiang Liu

**Affiliations:** 1 School of Biological Sciences, Nanyang Technological University, Singapore, Singapore; 2 Institute of Molecular and Cell Biology, Proteos, Singapore, Singapore; University of Hong Kong, Hong Kong

## Abstract

Coronavirus M protein is an essential component of virion and plays pivotal roles in virion assembly, budding and maturation. The M protein is integrated into the viral envelope with three transmembrane domains flanked by a short amino-terminal ectodomain and a large carboxy-terminal endodomain. In this study, we showed co-purification of the M protein from coronavirus infectious bronchitis virus (IBV) with actin. To understand the cellular factors that may be involved in virion assembly, budding and maturation processes, IBV M was used as the bait in a yeast two-hybrid screen, resulting in the identification of β-actin as a potentially interacting partner. This interaction was subsequently confirmed by coimmunoprecipitation and immunofluorescence microscopy in mammalian cells, and mutation of amino acids A159 and K160 on the M protein abolished the interaction. Introduction of the A159-K160 mutation into an infectious IBV clone system blocks the infectivity of the clone, although viral RNA replication and subgenomic mRNA transcription were actively detected. Disruption of actin filaments with cell-permeable agent cytochalasin D at early stages of the infection cycle led to the detection of viral protein synthesis in infected cells but not release of virus particles to the cultured media. However, the same treatment at late stages of the infection cycle did not affect the release of virus particles to the media, suggesting that disruption of the actin filaments might block virion assembly and budding, but not release of the virus particles. This study reveals an essential function of actin in the replication cycle of coronavirus.

## Introduction

Enveloped viruses acquire their envelope by budding from the host cell. In this process, viral envelope proteins gather at a special membranous structure and cooperate with other viral components to induce budding [1]. For example, some viruses including human immunodeficiency virus bud from the plasma membrane and release the virion from host cells by pinching-off. Others are budding at intracellular membranes [2], [3]. In this way, virions are wrapped within intracellular membrane-bound compartments, such as the endoplasmic reticulum (ER) and Golgi apparatus, and the newly budded viruses exit the cell by using the cellular secretory pathway [2]. However, the detailed mechanisms of viral assembly and budding, especially the host factors that are involved in these processes, are yet to be revealed for many viruses. In this study, we report that interaction between coronavirus membrane protein (M) and actin with functional implication in facilitating virion assembly and budding.

Coronavirus is an enveloped virus with a large, positive-stranded RNA genome of about 27 to 31 kilobases in length. The avian coronavirus Infectious bronchitis virus (IBV) belongs to the third group of coronaviruses genus. Like many other coronavriuses, IBV virion is built from four structural proteins, including the nucleocapsid (N) protein with which the genomic RNA is packed, the spike (S) protein that forms the prominent coronavirus spikes, the M protein which is the most abundant component of coronavirus, and the envelope (E) protein which is a minor but yet critical component in virion assembly [4]. Some group II coronaviruses also encode an additional structural protein, the hemagglutinin-esterase (HE).

Coronaviruses are known to assemble and bud at membranes of the intermediate compartment (IC), locating between the ER and Golgi complex [5]. The M protein is a type III membrane protein and a key player in coronavirus assembly. It spans the membrane bilayer three times, leaving a short amino-terminal domain on the virion exterior surface (or exposed luminally in intracellular membranes) and a large carboxy-terminal tail in the virion interior (or in the plasma) [6]. Lateral interactions between M proteins are thought to mediate the formation of the virion envelope [7]. When expressed alone, M protein accumulates in the Golgi complex in the form of homomultimeric complexes [8], [9]. However, in combination with the E protein, M is retained in the budding compartment and incorporated into virus-like particles (VLPs) with similarity in size and shape to authentic virions, demonstrating that the M and E proteins are the minimal requirements for envelope formation for most coronaviruses, [10]. The M protein appears to interact with S and HE proteins, and the S-M-HE protein complexes can be detected in cells infected with the bovine coronavirus [6]. The M protein was also shown to interact with the mouse hepatitis virus (MHV) nucleocapsid consisting of the genomic-size mRNA 1 and N protein in a pre-Golgi compartment, probably at the ER membrane. It may interact directly with the genomic RNA through the packaging signal, initiating the M-nucleocapsid interaction [11]. There is also a detectably direct interaction between M and N proteins in the nucleocapsid, which may further stabilize the M-genomic RNA interaction [11].

Actin is the most abundant protein in a typical eukaryotic cell, accounting for about 15% in some cell types [12]. The protein is highly conserved, differing by no more than 5% between species as diverse as algae and humans. It polymerizes in a helical fashion to form actin filaments (or microfilaments) that form the cytoskeleton, a three-dimensional network inside a eukaryotic cell. Actin filaments provide mechanical support for the cell, determine the cell shape, enable cell movements (through lamellipodia, filopodia, or pseudopodia), and participate in certain cell junctions, in cytoplasmic streaming and in cell contraction during cytokinesis [13].

In the present study, actin was shown to be co-purified with the IBV particles and was identified as a potential interacting protein of the IBV M protein. The interaction was subsequently confirmed by coimmunoprecipitation and immunofluorescent staining. Mutation of amino acids A159 and K160 in the M protein abolished the interaction. Introduction of the A159-K160 mutation into an infectious IBV clone system showed no infectious virus could be recovered, although viral RNA replication and subgenomic mRNA transcription were detected. Furthermore, treatment of the infected cells with cell-permeable agent cytochalasin D at early, but not late, stages of the replication cycle showed no release of the virion to the cultured media, suggesting that disruption of actin filaments might block virion assembly and budding.

## Materials and Methods

### Yeast two-hybrid screening

Yeast two-hybrid screening was performed with the pretransformed MATCHMAKER human bone marrow cDNA library (Clontech) according to the instructions of the manufacturer. In brief, the bait plasmid was transformed into yeast strain AH109, and transformants containing the bait plasmid were mated with the pretransformed cDNA library. Candidates were initially selected on SD-Ade/-His/-Leu/-Trp plates, and plasmid DNA was isolated from positive clones and sequenced according to the instructions of the manufacturer.

### Cell culture and transfection

H1299 and Vero cells were cultured in RPMI-1640 and complete Dulbecco's modified Eagle's medium (Invitrogen), respectively, supplemented with 10% new born calf serum (Sterile) and 1% penicillin/streptomycin (Invitrogen) and maintained at 37°C in humidified 5% CO_2._

Constructs containing plasmid DNA under the control of a T7 promoter were transiently expressed in mammalian cells using a vaccinia virus-T7 system. Briefly, 90% monolayers of H1299 cells were infected with 10 plaque forming units (PFU)/cell of the recombinant vaccinia virus (vTF-3), which express the T7 RNA polymerase gene, for 2 hours at 37°C prior to transfection. The plasmid DNA was transfected into vTF-3 infected cells using Lipofectamine 2000 reagent according to the instructions of the manufacturer (Invitrogen).

### Coimmunoprecipitaion

HeLa cells transfected with appropriate plasmids were lysed with TNTG lysis buffer (30 mM Tris-HCl pH 7.4, 150 mM NaCl, 1% NP40, and 10% glycerol) in the presence of 1×protease inhibitor mixture (Sigma) at 24 hours post-transfection. Total cell lysates were immunoprecipitated with appropriate antibodies for 2 hours at 4°C and further incubated for 2 hours at 4°C after adding buffer-balanced protein A agarose beads. The beads were washed three times and subjected to electrophoresis on SDS-12% polyacrylamide gel.

### Indirect immunofluorescence

IBV M protein was transiently expressed in H1299 cells grown on 4-well chamber slides (IWAKI). After rinsing with phosphate-buffered saline (PBS), cells were fixed with 4% paraformaldehyde for 15 minutes at room temperature and permeabilized with 0.2% Triton X-100, followed by incubating with specific antibodies diluted in fluorescence dilution buffer (PBS with 5% newborn calf serum) at room temperature for 2 hours. Cells were then washed with PBS, incubated with FITC-conjugated anti-rabbit secondary antibodies (DAKO) in fluorescence dilution buffer at 4°C for 1 hour and with Alexa Fluor 488 Phalloidin (Molecular Probe) at RT for 20 minutes before mounting. Confocal microscopy was performed on a Zeiss microscope.

### Western blot analysis

Samples were lysed with 2×SDS loading buffer and subjected to 10% SDS-PAGE. Proteins were transferred to PVDF membrane (Bio-Rad) by using a semi-dry transfer cell (Bio-Rad, Trans-blot SD), and blocked overnight at 4°C with 10% nonfat milk in PBS-T. The membranes were probed with specific primary antibodies followed by anti-mouse or anti-rabbit secondary antibodies conjugated with harderadish peroxidase (Sigma). Membrane-bound antibodies were detected with the enhanced chemiluminescence (ECL) detection reagents (Amersham, UK).

### Sucrose gradient purification of coronavirus

The virus was layered onto 20% TNE-buffered sucrose solution (TNE buffer: 50 mM Tris-Hcl, pH 7.4, 100 mM Nacl, 1 mM EDTA) and centrifuged at 175,000×g for 3 hours at 4°C (cushion). The resulting virus pellets were resuspended in TNE buffer, layered onto 10–50% linear sucrose gradient prepared with TNE buffer, and centrifuged at 175,000×g for 18 hours at 4°C. Aliquots of fractions starting from the top of the gradient were analyzed by SDS-PAGE.

### In vitro assembly and transcription of full-length cDNA clones

In vitro assembly and transcription of full-length cDNA clones were carried as previously described [14]. Briefly, plasmids were digested with either BsmBI (fragment A) or BsaI (fragments B, C, D and E). Bands corresponding to each of the fragments were cut from the gels and purified with QIAquick gel extraction kit (QIAGEN Inc.). All the fragments were ligated with T4 DNA ligase at 16°C overnight. The final ligation products were extracted with phenol/chloroform/isoamyl alcohol (25∶24∶1), precipitated with ethanol, and detected by electrophoresis on 0.8% agarose gels.

Full-length transcripts and N transcripts (using a linearized pKT0-IBVN containing IBV N gene and the 3′-UTR region as templates) were generated in vitro using the mMessage mMachine T7 kit (Ambion, Austin, Tex) according to the manufacturer's instructions.

### Electroporation of in vitro synthesized transcripts into Vero cells

Vero cells were grown to 90% confluence, trypsinized, washed twice with cold PBS, and resuspended in PBS (14). RNA transcripts were added to 400 µl of Vero cell suspension in an electroporation cuvette, and electroporated with one pulse at 450 V, 50 µF with a Bio-Rad Gene Pulser II electroporator. The transfected Vero cells were cultured overnight in 1% FBS-containing DMEM in a 60 mm dish and further cultured in DMEM without FBS.

### Reverse transcription, RT-PCR, and real-time PCR

Viral RNA was reverse-transcribed to cDNA using SuperScript III (Invitrogen) with modification to the protocol as follows: Random hexamers (300 ng) and total RNA (5 µg) were incubated for 10 minutes at 70°C. The remaining reagents were added according to the manufacturer's recommendation and the reaction was incubated at 55°C for 1 hour followed by 20 minutes at 70°C to inactivate the RT. For RT-PCR, a forward primer in the leader sequence and a reverse primer were used to generate a product by PCR. Quantitative real time RT-PCR was conducted using Smart Cycler II (Cepheid) with SYBR green (Cepheid) to detect subgenomic cDNA with primers (7.5 pM) optimized to detect a product spanning the leader sequence to the 5′ end of M gene or genomic cDNA with primers (7.5 pM). The cDNA from the RT reaction of each virus was diluted 1∶10, and 1 µl was used for each PCR, with a total reaction volume of 25 µl.

### Plasmid construction

M gene was cloned by digesting pIBVM with NcoI and EcoRI, and inserted into EcoRI/NcoI digested pGBKT7 to generate pGBKT7-M. The PCR fragment for actin was amplified by using primers 5′-CGCGGATCCATGGATGATGATATCGCCGCG-3′ and 5′-CCGCTCGAGGAAGCATTTGCGGYGGACGAT-3′. The PCR fragment was digested with BamHI and XhoI and ligated into BamHI and XhoI digested pXJ-myc to generate pXJ-myc-actin.

The deletion constructs MΔ1, MΔ2, MΔ3, MΔ4 and MΔ5 were made by two rounds of PCR as described previously [15]. The primer used for MΔ1 is 5′-AACTGCAGTTAAGCAAGCCACTGACCCTC-3′. The primers used for MΔ2 are 5′-TCTTTTGTAGGTTATAAGTGTGAACCAGAC-3′ and 5′-GTCTGGTTCACACTTA TAACCTACAAAAGA-3′. The primer for MΔ3 is 5′-AACTGCAGCCGCTTT GGTCACCAG-3′. The primers for MΔ4 are 5′-TGTGAGGGTCCAGACCACTTG-3′ and 5′-CAAGTGGTCTGGACCCTCACA-3. The primers for MΔ5 are 5′-GGTCAGTGGC TTTGTGAACCAGAC-3′ and 5′-GTCTGGTTCACAAAGCCACTGACC-3. All constructs were confirmed by automated nucleotide sequencing.

## Results

### Co-purification of actin with IBV

**Figure 1:**
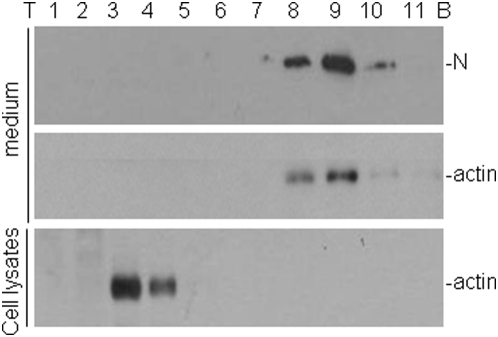
Co-Purification of actin with IBV virions. Confluent monolayers of Vero cells were infected with IBV at a multiplicity of infection of approximately 2 PFU/cell. At 18 hours post-infection, the supernatants were collected, centrifuged at 4,000 rpm for 20 minutes to remove cell debris. The virus was pelleted by ultracentrifugation through a 2 ml surcrose cushion (20%) at 175,000×g for 3 hours and further purified using a linear gradient of 10–50% sucrose at 175,000×g for 18 hours. Eleven fractions from top to bottom were collected and separated by SDS-PAGE. Polypeptides were analyzed by Western blot with anti-N antibodies (upper panel) and anti-actin antibodies (lower panel). Total lysates prepared from confluent monolayers of Vero cells were ultra-centrifuged at 175,000×g for 18 hours on a linear gradient of 10–50% sucrose. Eleven fractions from top to bottom were collected and separated by SDS-PAGE. Polypeptides were analyzed by Western blot with anti-actin antibodies.

In a previous study, it was observed that a cellular protein with migration properties on SDS-PAGE similar to actin was consistently co-purified with highly purified IBV particles [16]. However, as no suitable antibodies against actin were readily available at the time, the identity of this protein was not established. This issue was revisited in this study. Confluent monolayers of Vero cells were infected with IBV at a multiplicity of infection of approximately 2 PFU/cell. The supernatants were collected at 18 hours post-infection and centrifuged at 4,000 rpm for 20 minutes to remove cell debris. The virus particles were spun down by ultracentrifugation through a 2 ml sucrose cushion (20%), and purified using a 10–50% sucrose gradient. Following ultracentrifugation, 11 fractions were collected from top to bottom, and the presence of viral proteins was checked by Western blot with anti-N antibodies, showing that the IBV N protein was detected in fractions 7–11 with majority of the protein located in fractions 8–10 (Fig. 1, top panel). Analysis of the same fractions by Western blot with anti-actin antibodies showed that actin appeared in the same fractions as N protein, with the majority of the protein detected in fractions 8–10 (Fig. 1, middle panel). Fractionation of total lysates from cells without IBV infection under the same conditions showed that actin was mainly detected in fractions 3–4 (Fig. 1, bottom panel). These results demonstrate that actin could indeed be co-purified with the virus particles. However, it is currently uncertain if actin could be incorporated into the virions.

### Interaction of the IBV M protein with β-actin

In an attempt to search for IBV proteins that may interact with β-actin, IBV structural and nonstructural proteins were used as baits to screen a human cDNA library in yeast two-hybrid screening. Among all the IBV proteins used, only the C-terminal cytoplamsic portion of the IBV M protein was able to interact with β-actin.

**Figure 2:**
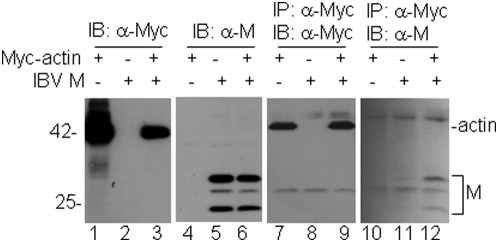
Interaction of the IBV M protein with human β-actin. HeLa cells were transfected with the Myc-tagged actin alone (lanes 1, 4, 7 and 10), IBV M alone (lanes 2, 5, 8 and 11) or cotransfected with actin and IBV M (lanes 3, 6, 9 and 12). Cells were harvested at 24 hours post-transfection and lysates prepared. Polypeptides were either analyzed directly by Western blot with anti-Myc (lanes 1–3) and anti-IBV M (lanes 4–6) antibodies, or subjected to immunoprecipitation with anti-Myc antibodies. The precipitates were analyzed by Western blot with anti-Myc (lanes 7–9) and anti-IBV M (lanes 10–12) antibodies. Numbers on the left indicate molecular masses in kilodaltons.

To confirm the interaction by co-immunoprecipitation, the full-length cDNA for β-actin was amplified by RT-PCR from HeLa cells, cloned into an expression vector with a c-Myc tag at its N-terminus (Myc-actin), and co-transfected into HeLa cells with the IBV M. Analysis of cells expressing the Myc-tagged actin either on its own or together with the M protein by Western blot with anti-Myc monoclonal antibody showed the detection of the Myc-tagged actin (Fig. 2, lanes 1 and 3). Similarly, analysis of cells expressing the M protein either on its own or together with the Myc-tagged actin by Western blot with anti-M polyclonal antibodies showed the detection of the full-length glycosylated (two upper bands) and unglycosylated (25 kDa) forms of the M protein (Fig. 2, lanes 5 and 6). The same cell lysates were then subjected to immunoprecipitation with anti-Myc antibody. Western blot analysis of the precipitates with the same anti-Myc antibody showed the detection of the Myc-tagged actin expressed either on its own or together with the M protein (Fig. 2, lanes 7 and 9). Western blot analysis of the same precipitates with anti-M antibodies showed the detection of the M protein only in cells co-expressing the two proteins (Fig. 2, lane 12). No detection of the M protein was found in cells expressing either the M protein or Myc-actin alone (Fig. 2, lanes 10 and 11). These results confirm that the IBV M protein could indeed interact with actin.

### Mapping of the interacting region in the M protein

**Figure 3:**
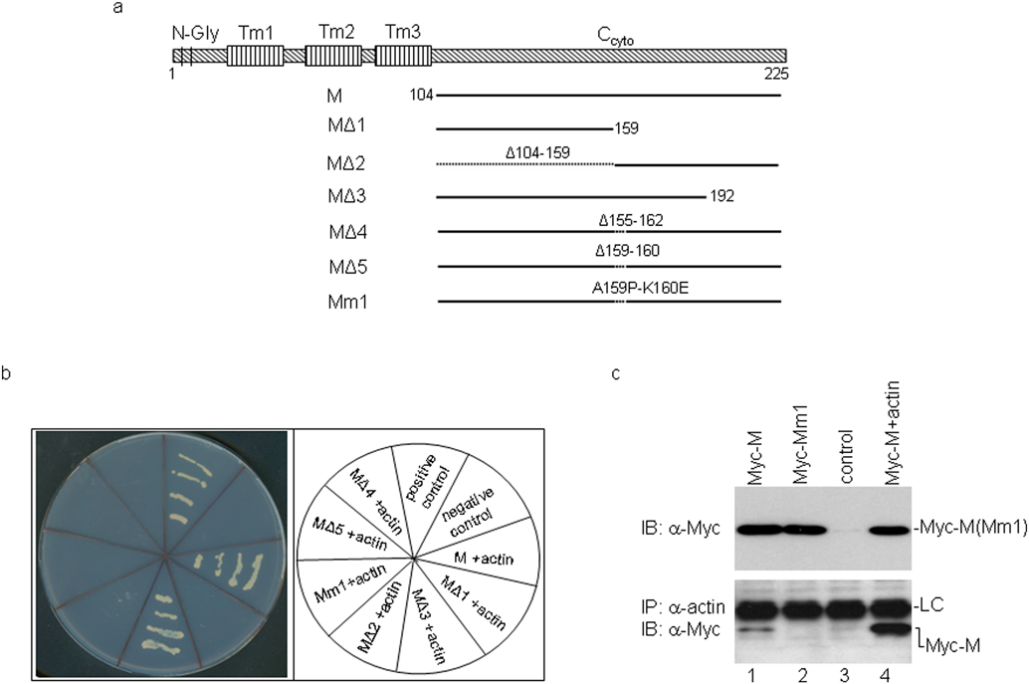
Mapping the region on M protein responsible for interaction with β-actin. a. Diagram showing the wild type IBV M and six deletion and mutation constructs (MΔ1, MΔ2, MΔ3, MΔ4, MΔ5 and Mm1). The two N-linked glycosylation sites (N-gly), three transmembrane domains (Tm1-3) and the C-terminal cytoplasmic domain of the M protein are highlighted. Also shown are the amino acid positions of the deleted and mutated region in each construct. b. The wild type IBV M and the six deletion and mutation constructs, respectively, were transformed into yeast cells with pACT-actin. The growth of the yeast cells on SD-Trp/-Leu/-His/-Ade- plate is shown. Also shown are the positive and negative controls. c. Co- immunoprecipitation of wild type M (the C-terminal region from amino acids 104–225) but not Mm1 with with human β-actin. HeLa cells were transfected with the Myc-tagged wild type M (Myc-M) (lane1), Myc-Mm1 (lane 2), empty vector (lane 3) or Myc-M+actin (lane 4). Cells were harvested at 24 hours post-transfection and lysates prepared. Polypeptides were either analyzed directly by Western blot with anti-Myc (upper panel), or subjected to immunoprecipitation with anti-actin antibodies. The precipitates were analyzed by Western blot with anti-Myc antibody (lower panel). LC-light chain.

To map the region in the M protein responsible for the interaction with actin by yeast two-hybrid screening, five deletion and one mutation constructs were made (Fig. 3a). In Figure 3a, MΔ1 contains the amino acid sequence from 104 to 159 (with deletion of amino acids 160 to 225); MΔ2 contains the deletion of amino acids 104–159; MΔ3 contains the amino acid sequence from 104 to 192 (with deletion of amino acids 193 to 225); MΔ4 contains the deletion of amino acids 155–162; and MΔ5 contains the deletion of amino acids 159–160 (Fig. 3a). The mutant construct Mm1 contains the mutation of amino acids 159 and 160 from alanine and lysine to proline and glutamic acid, respectively (Fig. 3a). Introduction of individual deletion and mutant constructs into the yeast stain AH109 together with pACT-actin showed growth of wild type and MΔ3 constructs on SD-Trp/-Leu/-His/-Ade selective plates (Fig. 3b). However, none of the other deletion and mutant constructs could grow on the same plate (Fig. 3b). Western blot analysis showed similar expression levels of wild type and mutant constructs in yeast (data not shown). As MΔ5 contains the minimal deletion of two amino acids, this study maps the actin-binding site on the M protein to the region containing amino acids A159 and K160.

Co-immunoprecipitation was then carried out by expressing the Myc-tag wild type M (C-terminal domain from amino acid 104 to 225, Myc-M) and Myc-Mm1 in cells. Western blot analysis with anti-Myc monoclonal antibody showed the expression of Myc-M and Myc-Mm1 at a similar level (Fig. 3c, upper panel, lanes 1 and 2). The same cell lysates were then subjected to immunoprecipitation with anti-actin antibody, and Western blot analysis of the precipitates with anti-Myc antibody showed the presence of Myc-M but not Myc-Mm1 (Fig. 3c, lower panel, lanes 1 and 2). The detection of Myc-M in the immunoprecipitates was greatly enhanced by co-expression of Myc-M and actin (Fig. 3b, lane 4).

### Colocalization of the M protein with actin in cells treated with cytochalasin D

**Figure 4:**
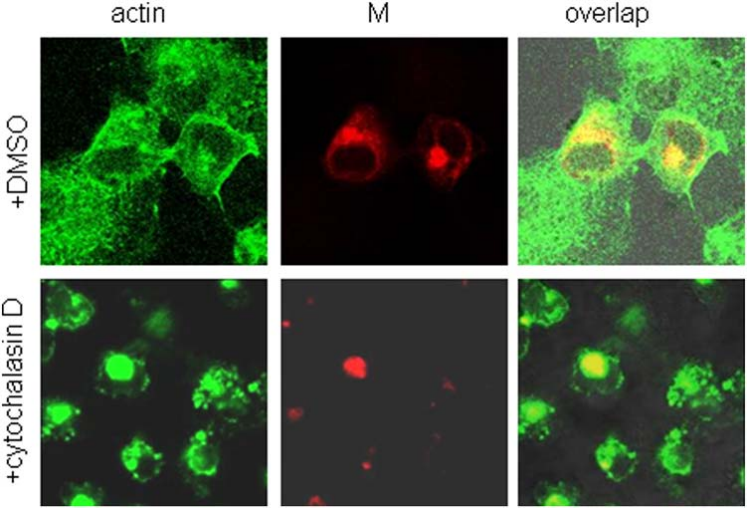
Colocalizaion of M and actin proteins in cells. The plasmids containing M gene were transfected into H1299 cells. At 4 hours post-transfection, the cells were treated by cytochalasin D (12.5 µg/ml) and permeabilized with 0.2% Triton X-100 and stained with anti-M antibodies at 48 hours post-transfection. The same cells were also stained with actin dye Alexa Fluor 488 Phalloidin (green).

As a type III membrane protein with three transmembrane domains, the IBV M protein is mainly localized to the Golgi apparatus in virus-infected cells and in cells expressing the M protein [8]. Cytochalasin D is a cell-permeable fungal toxin. It can bind to the barbed end of actin filaments and inhibit both the association and dissociation of actin subunits, resulting in the disruption of actin filaments and inhibition of actin polymerization. To test the subcellular localization of the M protein in cells treated with cytochalasin D, H1299 cells were transfected with M construct and 12.5 µg/ml of cytochalasin D was added to the cells at 4 hours post-transfection. Cells were permeabilized with 0.2% Triton X-100 and stained with anti-M antibodies at 48 hours post-transfection. The same cells were also stained with Alexa Fluor 488 Phalloidin, a specific dye for actin (Molecular Probe). Staining of cells treated with DMSO and Alexa Fluor 488 Phalloidin showed a normal three-dimensional network (Fig. 4, upper panels). Staining of cells treated with cytochalasin D and Alexa Fluor 488 Phalloidin showed that the regular cell actin filaments were destroyed (Fig. 4, lower panels), displaying random distribution of actin dots in cells without the M protein expression. In cells expressing the M protein, both M protein and actin were found mainly in the Golgi area with fairly well overlapping of the two staining patterns (Fig. 4, lower panels).

### Effects of deletion and mutation of amino acid residues A159 and K160 on the replication, budding and release of IBV

Two approaches were used to test the effect of deletion and mutation of amino acid residues A159 and K160 in the M protein on the replication, budding and release of IBV. First, the A159-K160 deletion and mutation were introduced into a full-length IBV infectious clone, and the effect of this minimal deletion and mutation on the replication of viral RNA and the recovery of infectious IBV was analyzed. Introduction of full-length transcripts derived from wild type (wt), the A159-K160 deletion construct (MΔ5) and A159-P/K160-E mutation construct (Mm1) into Vero cells by electroporation showed efficient recovery of infectious IBV from cells transfected with wild type transcripts. However, it was consistently observed that no infectious virus could be recovered from cells transfected with either MΔ5 or Mm1 transcripts.

**Figure 5:**
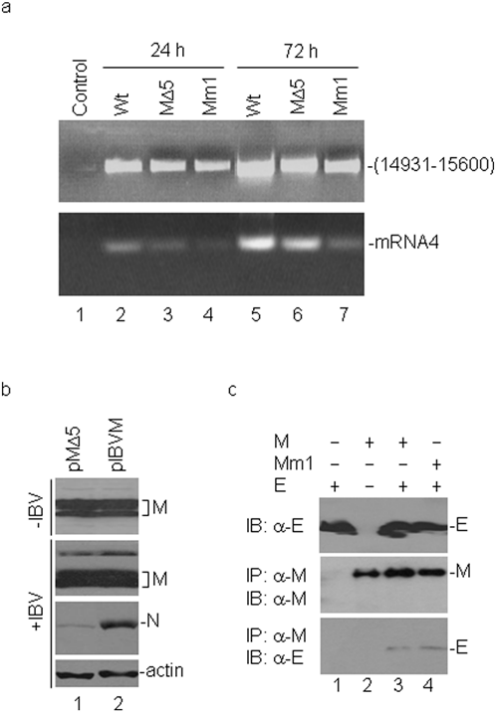
Effects of A159-K160 deletion and mutation on replication and infectivity of IBV. a. In vitro transcripts derived from wild type, MΔ5 and Mm1 full-length IBV clones were electroporated into Vero cells. At 24 and 72 hours post-electroporation, total RNA were extracted from the cells and specific primers were used to detect the minus RNA (upper panel) and subgenomic mRNA 4 (lower panel) by RT-PCR. b. H1299 cells expressing wild type IBV M and MΔ5 constructs with a vaccinia virus system were infected with IBV at a multiplicity of infection of approximately 0.5 PFU/cell. Cells were collected at 24 hours post-infection and polypeptides were analyzed by Western blot with anti-IBV M (second panel), anti-IBV N (third panel) and anti-actin (bottom panel) antibodies. H1299 cells expressing wild type IBV M and MΔ5 constructs with a vaccinia virus system without IBV infection harvested at 40 hours post-transfection were also analyzed by Western blot with anti-IBV M antibodies (top panel). c. Interaction of Mm1 mutant M protein with IBV E protein. H1299 cells expressing the E (lane 1), M (lane 2) and E+M (lane 3) and E+Mm1 were harvested at 24 hours post-transfection and lysed. The total cell lysates were either detected directly by Western blot with anti-E antibodies (top panel) or immunoprecipitated with anti-M antibodies. The precipitates were analyzed by Western blot with anti-M (middle panel) and anti-E (bottom panel) antibodies, respectively.

As no infectious virus was recovered from cells transfected with MΔ5 and Mm1 transcripts, RT-PCR amplification of the negative strand RNA was performed to check if RNA replication occurred in the transfected cells. The primer pair (5′-^14931^GCTTATCCACT AGTACATC^14949^-3′ and 5′-^15578^CTTCTCGCACTTCTGCACTAGCA^15600^-3′) was chosen to amplify the negative strand IBV sequence from nucleotides 14931 to 15600 by the RT-PCR. If replication of viral RNA occurred, a 670 bp PCR fragment would be expected. As shown in Fig. 5a, the 670 bp RT-PCR fragments were obtained from cells transfected with both wild type and mutant transcripts at 24 and 72 hours post-transfection, respectively (upper panel, lanes 2–7). Interestingly, the amounts of the fragment detected from cells transfected with MΔ5 and Mm1 transcripts were increased at 72 hours post-transfection (Fig. 5a, upper panel, lanes 3–4 and 6–7), demonstrating the replication of the transfected mutant transcripts. No RT-PCR fragment was detected in cells without transfection (Fig. 5a, upper panel, lane 1).

RT-PCR amplification of subgenomic mRNAs was then carried out to check if subgenomic mRNA synthesis could occur in cells transfected with the mutant transcripts at 24 and 72 hours post-electroporation. The forward primer (5′-ACTTAAGATAGATATTA A-3′) used in this reaction corresponds to the leader sequence from nucleotides 26–46 in the genomic RNA and the downstream primer (5′-CTCTGGATCCAATAACCTAC-3′) covers the IBV sequence from nucleotides 24784 to 24803. If transcription of subgenomic mRNAs occurs, a 415 bp PCR product corresponding to the 5′-terminal region of the subgenomic mRNA4 would be detected. As shown in Fig. 5a, a dominant 415 bp was observed in cells electroporated with wild type full-length transcripts at two time points (lower panel, lanes 2 and 5). In cells transfected with MΔ5 and Mm1 transcripts, a much weaker band was detected in cells transfected with the two mutant transcripts at 24 hours post-transfection (Fig. 5a, lower panel, lanes 3–4). Similarly to the results described above, the amounts of the subgenomic RNA 4 fragment detected from cells transfected with MΔ5 and Mm1 transcripts were increased approximately 3 to 5 fold, respectively, at 72 hours post-transfection based on real time PCR assay (Fig. 5a, lower panel, lanes 3–4 and 6–7). As a negative control, no detection of the amplified fragments was obtained from a mixture of RNA templates containing the *in vitro* transcribed RNA and total RNA extracted from mock infected cells (Fig. 5a, lower panel, lane 1).

In the second approach, plasmids containing wild type M (pIBVM) and the A159-K160 deletion M sequences (pMΔ5) were transfected into H1299 cells using the vaccinia/T7 recombinant virus expression system. H1299 cells are a continuous cell line derived from human lung carcinoma. This cell line can be efficiently infected by the Vero cell-adapted IBV [17] and is much more resistant to the vaccinia virus-induced morphological changes. At 16 hours post-transfection, the cells were infected with IBV at a multiplicity of infection of approximately 0.5 PFU/cell, and were harvested at 24 hours post-infection. The expression of M protein and the infectivity of IBV on these cells were analyzed by Western blot with anti-M and anti-N polyclonal antibodies, respectively (Fig. 5b). In cells transfected with both wild type and MΔ5 constructs with or without IBV infection, detection of similar amounts of M protein was observed (Fig. 5b, top two panels, lanes 1 and 2), suggesting that both constructs were expressed at similar efficiencies. However, much more N protein was detected in the infected cells transfected with wild type M construct than that in cells transfected with the MΔ5 mutant (Fig. 5b, middle panel, lanes 1 and 2). As a loading control, similar amounts of actin were detected in cells transfected with both constructs (Fig. 5b, bottom panel). These results confirm that expression of the A159-K160 deletion M protein significantly reduces the infectivity of IBV, suggesting that expression of this mutant protein could inhibit the production of infectious virus in a dominant-negative manner.

To rule out the possibility that the deletion and mutation may render detrimental effect on the overall structure of the M protein and its interaction with other viral proteins that are essential for virion assembly, interaction of the Mm1 mutant with the E protein was tested by co-immunoprecipitation. As shown in Fig. 5c, Western blot analysis with anti-IBV E protein antibodies showed the presence of E protein in cells expressing the protein on its own or together with wild type or Mm1 mutant protein (top panel). Immunoprecipitation of the same lysates with anti-M antibodies and subsequent analysis of the precipitates by Western blot with anti-M antibodies led to the detection of wild type or the Mm1 mutant proteins in cells expressing M forms either alone or together with the E protein (Fig. 5c, middle panel). Western blot analysis of the same precipitates with anti-IBV E antibodies, however, showed the presence of E protein only if it was co-expressed with either wild type or the Mm1 mutant protein (Fig. 5c, bottom panel). These results confirm that the A159-P/K160-E mutations did not affect the interaction between IBV M and E proteins.

### Effects of disruption of actin filaments by cytochalasin D on the replication, budding and release of IBV

**Figure 6:**
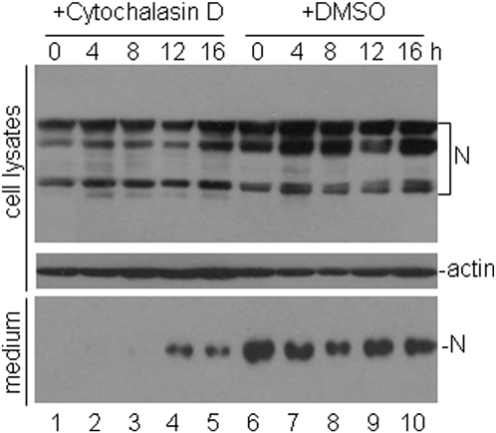
Effects of disruption of actin filaments by cytochalasin D on IBV replication. Vero cells plated in six-well plates were infected with IBV at a multiplicity of infection of approximately 3 PFU/cell. Cytochalasin D (12.5 µg/ml) and DMSO were added to the infected cells at indicated time. Total cells and the culture media were separately collected at 24 hours post-infection. Cell lysates were prepared and analyzed by Western blot with anti-N (top panel) and anti-actin (middle panel) antibodies. Western blot analysis of the IBV N protein in the culture media with anti-N antibodies (bottom panel) was also shown.

To define more precisely the stage of the viral replication cycle that is facilitated by the interaction between M protein and actin, the effects of disrupting actin filaments by cytochalasin D on the replication, budding and release of IBV were tested by detailed time-course experiments. Confluent monolayers of Vero cells in six-well plates were infected with IBV at a multiplicity of infection of approximately 3 PFU/cell. At 0, 4, 8, 12 and 16 hours post-infection, either 12.5 µg/ml of cytochalasin D or equal volume of DMSO was added to the cells. The supernatants and cell pellets were separately harvested at 24 hours post-infection and the presence of IBV N protein was checked by Western blot with anti-N antibodies. Typical N protein profiles, including the full-length and posttranslationally modified forms of the protein, were detected in the total cell lysates (Fig. 6, top panel). In cells treated with cytochalasin D, slightly less amounts of the N protein were detected when cytochalasin D was added to the cells at 0, 4, 8 and 12 hours post-infection, respectively (Fig. 6, top panel, lanes 1–4). No obvious difference in the expression of N protein was seen when cytochalasin D was added at 16 hours post-infection (Fig. 6, top panel, lane 5). Western blot analysis of actin was included as a loading control (Fig 6, middle panel). Analysis of the N protein in the clarified supernatants showed the detection of a single N protein species that represents the N protein incorporated into the virion particles in cells treated with DMSO alone (Fig. 6, bottom panel, lanes 6–10). In supernatants collected from cells treated with cytochalasin D, much reduced amounts of the N protein were detected in cells treated with the reagent at 12 and 16 hours post-infection, respectively (Fig. 6, bottom panel, lanes 4 and 5). No N protein was detected in supernatants harvested from cells treated with cytochalasin D at 0, 4 and 8 hour post-infection, respectively (Fig. 6, bottom panel, lanes 1–3).

## Discussion

Coronavirus M protein plays essential roles in virion assembly and budding [18], [19]. The protein itself cannot bud. However, with the help of E protein, VLPs may be formed at the IC membranes. As coronaviruses do not contain a matrix protein that underlines the membrane, the M protein may serve as a matrix-like protein. It is proposed that the formation of coronavirus envelope is dominated by lateral interaction between M molecules that form a two-dimensional lattice in intracellular membranes [20]. The M-M interaction is essential but not sufficient for coronavirus envelope assembly. Free energy is needed to generate and stabilize membrane curvature, suggesting that interaction of M protein with host proteins would be a must [21]. To support this speculation, the results present in this study demonstrate that interaction of the IBV M protein with β-actin is essential for virion assembly and budding. The interacting region in the M protein was pinpointed by deletion and mutation studies. As MΔ3 is able to interact with actin, but MΔ1 and MΔ2 could not, it was reasoned that the domain responsible for this interaction may be located in the region covering amino acids 159–160. This region was chosen for further deletion and mutational analysis, confirming that it is indeed involved in the interaction with actin. However, the two amino acids are not conserved in other coronavirus M proteins. At present, we do not know whether interaction between coronavirus M protein and actin is a general phenomenon for all coronaviruses, or is just IBV-specific.

Available evidence suggests that the host cytoskeleton, especially actin, is involved in the budding process of several animal viruses [22]. Actin has been identified in many enveloped viruses, such as Rous sarcoma virus, mouse mammary tumor virus, Sendai virus and measles virus. In the case of measles virus, actin was originally thought as a cellular contaminant, but was later demonstrated to be an internal component of the virus [23]–[25]. Actin was found in the purified paramyxovirus particles and actin filaments were seen in association with the budding virions by electron microscopy [24]–[26]. Polymerized actin was also found in HIV preparations [27], [28]. The functional implication for the incorporation of actin into these virus particles remains unclear. One possibility is that actin polymerization serves as an additional force for membrane bending during budding [1], [29]. As an illustrative example, actin was shown to control the movement of measles virus envelope proteins on the surface of infected cells [30].

Interaction of actin with viral components was also reported in several other viral systems. One example is the interaction of the nuclocapsids of murine mammary tumor virus with actin, which was required for extruding the virus particles from virus-infected cells [31]. The M protein of Newcastle disease virus could interact with actin in vitro [1], [32]. The SARS-CoV N protein could aggregate elongation factor 1α through direct interaction, leading to the inhibition of protein translation and cytokinesis by blocking F-actin bundling [33]. It was also reported that the SARS-CoV N protein could induce apoptosis and actin reorganization in mammalian cells under stress conditions [34]. The results shown in this study demonstrate that actin could be co-purified with IBV virions and that the IBV M protein is associated with actin, suggesting that actin is likely to be incorporated into IBV virions through its interaction with the M protein. However, more conclusive data, such as immunogold labeling of highly purified virions, are currently lacking. It is interesting that IBV replication cycle was disturbed in cells treated with cytochalasin D. Cytochalsin D could prevent actin from polymerization, leading to malfunction of actin. When actin was disrupted, much less IBV virions were released from the cells into the culture medium, demonstrating that actin plays a very important role in the IBV replication cycle. Two possible mechanisms were considered. First, actin may be involved in the formation of the M lattice that may promote and stabilize its curving, resulting in the enhancement of some late processes, such as virion budding and release. Second, the MHV M protein was shown to be recycled from the Golgi complex back to early compartments, such as the ER and IC, which are functionally important in coronavirus infected cells [21]. Therefore, interaction of actin with M protein may help the retrograde transport of M protein from Golgi apparatus to some early compartments and provide more M protein for virion assembly and budding.

The actin-binding proteins are grouped into 60 distinct classes based on primary structures [35]. The sequence of the actin-binding sites ranges from 10–30 residues, which shows no obvious homology among different classes [36]. For example, Aldolase, a glycolytic enzyme, binds actin filaments to concentrate enzyme and substrate [37]. The sequence 32-ADESTGSIAKRLQSIGTENTE-52 of aldolase has been identified as the actin-binding motif [38]. Furthermore, Carcinoembryonic Cell Adhesion Molecule 1 (CEACAM1) was found to bind F-actin. The actin binding site is FLHFGKTGSSGPLQ, which is not similar to other existing actin-binding sequences [39].

Coronavirus M protein is an essential and predominant component of the virions. It plays pivotal roles in virion assembly and budding by interaction with other viral components. Firstly, the monomers of M could interact with each other particularly via the transmembrane domains [21]. Besides, the M-N interaction region was narrowed to the 16 residues adjacent to the carboxy terminus of TGEV M protein [40]. However, the specific amino acids on M protein responsible for M-S and M-E interactions are still enigmatic. In the present study, deletion or mutation of amino acids A159 and K160, which are essential for the interaction of M protein with actin, is detrimental to the recovery of IBV from an infectious IBV clone due to a defect in late stages of the IBV replication cycle. Although, the deletion or mutation of amino acids A159 and K160 could also interact with another structural protein E, we cannot exclude the possibility that this region is essential for maintaining the integrity of the M protein and involved in the interactions with other viral components. Nevertheless, it lends support to the conclusion that interaction of the IBV M protein with actin is essential for the completion of the IBV infection cycle.

More evidence is derived from the study using cytochalasin D. Similar amount of viral proteins were detected in the total cell lysates but were not detected in supernatants when cytochalasin D was added early in the viral replication cycle suggests that actin filaments are essential for some early events, such as virion assembly and budding, during the assembly and maturation processes of IBV. As IBV proteins can be efficiently detected in the supernatants when cytochalasin D was added at 12 and 16 hours post-infection, respectively, it suggests that actin filaments are unlikely involved in the release of the IBV particles. Instead, our data support that actin filaments may be involved in the virion assembly and budding process of the coronavirus replication cycles. As coronavirus M protein could bind to the viral genomic RNA through packaging signal [41] and RNA replication in the cytoplasm would rely on the support of actin [42], the interaction of M protein with actin may serve as a platform to promote the binding of M protein to viral RNA as well as viral RNA replication. Alternatively, coronavirus M protein could bind to the N protein-RNA complex to stabilize the nucleocapsid [11] and extra energy would be needed in this process [21]. The interaction of M protein with actin may serve as a power station to provide energy for completion of the viral replication cycle.
